# Optimal design of cluster randomized crossover trials with a continuous outcome: Optimal number of time periods and treatment switches under a fixed number of clusters or fixed budget

**DOI:** 10.3758/s13428-024-02505-1

**Published:** 2024-09-13

**Authors:** Mirjam Moerbeek

**Affiliations:** Department of Methodology and Statistics, Utrecht University, PO Box 80140, Utrecht, TC 3508 The Netherlands

**Keywords:** Cluster randomized trial, Crossover design, Optimal design, Budgetary constraint, Treatment switches, Relative efficiency

## Abstract

In the cluster randomized crossover trial, a sequence of treatment conditions, rather than just one treatment condition, is assigned to each cluster. This contribution studies the optimal number of time periods in studies with a treatment switch at the end of each time period, and the optimal number of treatment switches in a trial with a fixed number of time periods. This is done for trials with a fixed number of clusters, and for trials in which the costs per cluster, subject, and treatment switch are taken into account using a budgetary constraint. The focus is on trials with a cross-sectional design where a continuous outcome variable is measured at the end of each time period. An exponential decay correlation structure is used to model dependencies among subjects within the same cluster. A linear multilevel mixed model is used to estimate the treatment effect and its associated variance. The optimal design minimizes this variance. Matrix algebra is used to identify the optimal design and other highly efficient designs. For a fixed number of clusters, a design with the maximum number of time periods is optimal and treatment switches should occur at each time period. However, when a budgetary constraint is taken into account, the optimal design may have fewer time periods and fewer treatment switches. The Shiny app was developed to facilitate the use of the methodology in this contribution.

## Introduction

The cluster randomized trial, in which complete clusters such as schools, general practices, and households are randomized to treatment conditions, is common in social, health, and medical science (Campbell & Walters, 2014; Donner & Klar, 2000; Eldridge & Kerry, 2012; Hayes & Moulton, 2022; Murray, 1998). Within each cluster, a certain number of subjects is enrolled, treated, and measured. This trial design is often chosen for political, ethical, and financial reasons (Gail et al., 1996) and the need to avoid contamination of the control condition (Hemming et al., 2021; Moerbeek, 2005). Cluster randomization comes at a price, though, namely a decreased efficiency as compared to a design with individual randomization and the same number of subjects. This follows from the fact that once subjects are nested within clusters, their responses become more similar due to mutual influence, cluster policy, and cluster norms. As a result, the effective sample size is smaller than the actual sample size and hence the efficiency decreases.

**Fig. 1:**
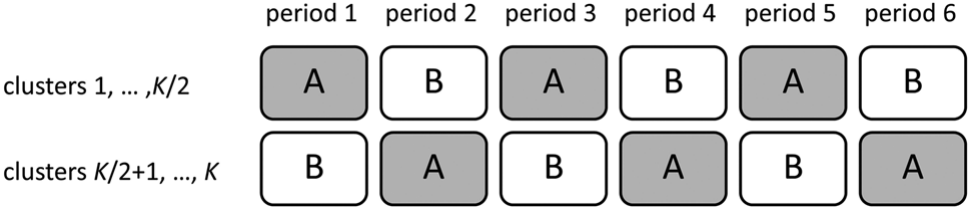
Graphical representation of a cluster randomized crossover trial with six time periods and two treatment sequences. Clusters alternate between treatments A and B with each time period. The same number of clusters $K/2$ is enrolled in each treatment sequence

**Fig. 2:**
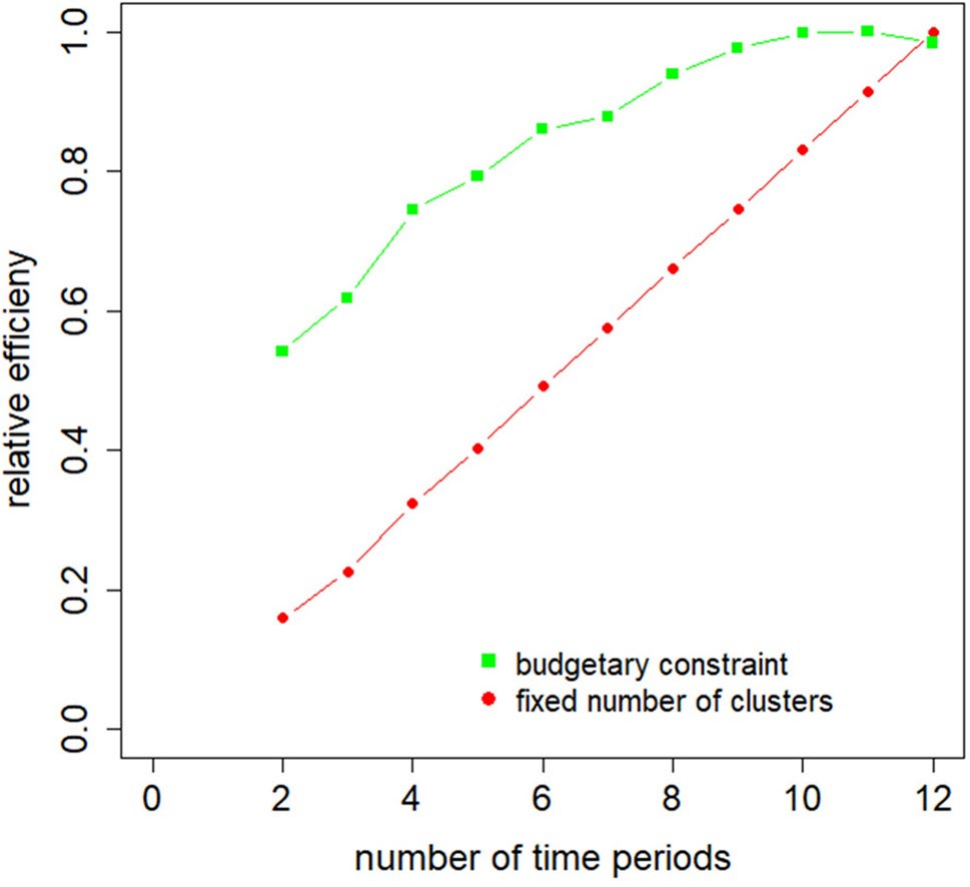
Relative efficiency of the cluster randomized crossover trial as a function of the number of time periods, for trials with a fixed number of clusters (*top line*) or a budgetary constraint (*bottom line*)

One approach to compensate for the loss of efficiency is implementing a crossover design (Brown, 1980; Jones & Kenward, 2002; Senn, 2002) within a cluster randomized trial. Clusters are then randomized to a sequence of treatment conditions, rather than just one single treatment condition. Clusters thus alternate between two or more treatments in a trial that includes at least two time periods. A time period is a period in time in which a treatment is implemented. Within each time period and each cluster, a certain number of subjects is enrolled and treated, and at the end of each time period outcome measures are taken on those subjects who are enrolled in that time period. This is illustrated in Fig. 1, in which $K$ clusters alternate between treatment conditions A and B in a trial of six time periods. $K$ can be any even integer $\ge 2$ and the value of $K$ does not depend on the number of time periods. The first half of clusters starts with treatment A, then switches to B, switches back to A, and so forth. This treatment sequence ABABAB is the one in the first row of Fig. 2. The second half of clusters starts with treatment B, then switches to A, and so forth. This treatment sequence BABABA is the one in the second row of Fig. 2. We can consider the one treatment sequence to be a mirror of the other treatment sequence as within each time period they have different treatments, but both of them consist of six time periods. At the end of the study, each cluster was involved in six time periods, three time periods with treatment condition A and another three with treatment condition B. As both conditions are available within each cluster, the crossover design is a within-cluster design. Hence, each cluster serves as its own control and this increases the efficiency of the design.

Over the past years, various papers that deal with the optimal design and power analysis of the cluster randomized crossover trial have been published (Arnup et al., 2017; Giraudeau et al., 2008; Li et al., 2019; Moerbeek, 2020; Rietbergen & Moerbeek, 2011). The main focus of these papers is on the so-called AB/BA design, in which half of the clusters receive treatment A in time period 1 and treatment B in time period 2, and the other half of the clusters receive these treatments in reverse order. This is the simplest crossover design as it has only two time periods. It is, however, very well possible to include more than two time periods; some clinical trials have included as many as 12 time periods (Charehbili et al., 2019; Semler et al., 2017; Spence et al., 2018). This raises the question of what the optimal number of time periods in a cluster randomized trial is. This question has already been studied for trials with fixed study duration, fixed number of subjects per cluster and continuous recruitment (Grantham, Kasza, Heritier, Hemming, Litton et al., 2019b). Increasing the number of time periods in such a setting implies a shorter duration of each time period and hence fewer subjects per time period. Such a setting may not always be realistic in practice. In group behavioral interventions, treatment is often offered in a time period of fixed duration with a pre-set number of sessions over a period of some weeks or months. This often applies to psychotherapy trials and health prevention and education interventions as well. Also in clinical trials, there may be reasons from a logistical or administrative nature to use time periods of fixed duration. For instance, the equipment used in a certain treatment condition, or replaceable components thereof, may have a lifespan of limited duration and it may be considered cost-inefficient to switch treatments before the maximum lifespan has been reached. Furthermore, the number of subjects that can be treated in a certain time period is often also limited. For instance, the number of participants in a group behavioral intervention may be fixed to facilitate group discussion. Also, the number of subjects that can be treated by a health professional or by using some equipment may be limited.

The first aim of this contribution is to study the optimal number of time periods in the treatment sequences in a cluster randomized crossover trial. The focus is on studies where time periods have a fixed duration and a fixed number of subjects can be treated per time period per cluster. So the optimal design problem is different from the one by Grantham, Kasza, Heritier, Hemming, Litton et al. (2019b), where the duration of the study was fixed. Increasing the number of time periods in the present paper implies increasing study duration and increased number of subjects per cluster. One may expect to include more time periods in a treatment sequence to result in a more efficient design, but increasing the number of time periods may be disadvantageous when costs are associated with each treatment switch. A budgetary constraint will therefore be taken into account when deriving the optimal number of time periods.

A second aim of this contribution is to study if treatment switches should occur with each new time period. In some trials, it may not be necessary to alternate between treatments A and B at each new time period. Especially when the costs of a treatment switch are relatively high, then a design in which clusters stay in the same treatment across a few consecutive time periods may be more efficient.

The focus is on cross-sectional trials, meaning that each subject is enrolled and treated in only one time period and thus receives either treatment A or B but not both. This is in contrast to the cohort design, where subjects are enrolled and treated in all time periods and hence receive both treatments A and B. The measurement scheme is discrete-time: each subject’s outcome is measured at the end of the time period in which he or she is enrolled and treated. A linear multilevel (mixed effects) model is used to model the relation between time period, treatment condition, and a quantitative response, thereby using an exponential decay correlation structure. Correlations between two subjects within the same cluster become smaller if the time periods in which these subjects are enrolled are further away in time. Alternative designs are compared on their variance of the treatment effect estimator, taking into account the costs of each design. For both research aims, the optimal design, with the smallest variance of the treatment effect estimator, will be identified and the efficiency of alternative designs will be calculated to identify highly efficient designs, which perform nearly as well as the optimal design. The methodology will be illustrated using a trial to reduce occupational sedentary behavior (i.e., sitting for too much time) and is implemented in the Shiny app.

## Optimal design methodology

### Statistical model

The multilevel model (Goldstein, 2011; Hox et al., 2018; Snijders & Bosker, 2012), also known as a hierarchical model (Raudenbush & Bryk, 2002) or mixed effects model, is used to specify the relation between a subject’s outcome variable and the treatment condition he/she received and the time period in which he/she was enrolled. Measurements are taken at the end of each time period (i.e., at the end of treatment or therapy or the control).

The model for subject $i=1,\dots ,m$ in time period $t=1,\dots ,T$ in cluster $k=1,\dots ,K$ is

$${Y}_{kti}=\mu +{\beta }_{t}+\theta {X}_{kt}+{CP}_{kt}+{\varepsilon }_{kti},$$

where ${Y}_{kti}$ is the quantitative outcome, $\mu$ is the overall mean outcome, ${\beta }_{t}$ is the fixed effect of time period $t=2,\dots ,T$ (with ${\beta }_{1}=0$ for identifiability^1^), ${X}_{kt}$ is the treatment indicator (with values 0 and 1 for the control and intervention condition, respectively), $\theta$ is the fixed treatment effect, ${CP}_{kt}\sim N\left(0,{\sigma }_{CP}^{2}\right)$ is the random cluster-period effect and ${\varepsilon }_{kti}\sim N\left(0,{\sigma }_{\varepsilon }^{2}\right)$ is the random individual-level error. The first is the deviation of the mean outcome in cluster $k$ in time period $t$ from the mean outcome in that time period across all clusters in the same treatment sequence. The latter is the deviation of the outcome of subject $i$ from the mean outcome in cluster $k$ in time period $t$. The intraclass correlation coefficient, which is calculated as $\rho =\frac{{\sigma }_{CP}^{2}}{{\sigma }_{CP}^{2}+{\sigma }_{\varepsilon }^{2}}\in \left[\text{0,1}\right]$, quantifies the proportion variance at the cluster level. A discrete-time exponential decay correlation structure (Grantham, Kasza, Heritier, Hemming, & Forbes, 2019a; Kasza et al., 2019) is used to allow for the correlation of two subjects within the same cluster to decrease with increasing time lag between these subjects: $cov\left({CP}_{kt},{CP}_{k{t}^{^\prime}}\right)={\sigma}_{CP}^{2}{r}^{\left|t-{t}^{^\prime}\right|}$, with $r\in \left[\text{0,1}\right]$. The latter parameter is called the cluster autocorrelation, which is assumed constant across clusters and cluster-periods and does not depend on the number of time periods in a treatment sequence either. The smaller its size, the lower the correlation between outcomes of subjects within the same cluster and hence the lower the variance of the treatment effect estimator. Its complement $1-r$ is the decay in the correlation between any two adjacent time periods. So, the outcomes of subjects who are measured further apart in time have a lower correlation than the outcomes of subjects who are measured closer in time. The decay in correlation between outcomes in the first and last time period is calculated as $1-{r}^{T-1}$ and hence becomes larger if the number of time periods in a treatment sequence increases. For instance, if $r=0.9$ then the decay in correlation between the first and last time period is $1-{0.9}^{2}=0.19$ in a study with three time periods and $1-{0.9}^{11}=0.69$ in a study with 12 time periods.

The model can also be formulated in terms of cluster-period means (Kasza et al., 2019, 2020). The mean outcome in time period $t$ cluster $k$ is calculated as

$${\overline{Y} }_{kt.}=\frac{1}{m}\sum\limits_{i=1}^{m}{Y}_{kti}.$$

It is modeled as

$${\overline{Y} }_{kt.}={\mu +\beta }_{t}+\theta {X}_{kt}+{CP}_{kt}+{\overline{\varepsilon }}_{kt.}.$$

The parameters $\mu$, ${\beta }_{t}$, $\theta$, treatment indicator ${X}_{kt}$ and random effect ${CP}_{kt}$ are as above. The random effect

$${\overline{\varepsilon }}_{kt.}=\frac{1}{m}\sum\limits_{i=1}^{m}{e}_{kti}$$

is the mean individual-level error in cluster-period $t$ in cluster $k$, which is assumed to be normally distributed with zero mean and variance $var\left({\overline{\varepsilon }}_{kt.}\right)=\frac{{\sigma }_{\varepsilon }^{2}}{m}.$

The $T\times T$ covariance matrix of the $T$ mean responses in cluster $k$ is denoted $V$ and is defined as $V=\frac{{\sigma }_{\varepsilon }^{2}}{m}I+{\sigma }_{CP}^{2}R$, where $I$ is the identity matrix and matrix $R$ has value 1 on the diagonal and value ${r}^{|t-t^{^\prime}|}$ in row $t$ and column $t^{^\prime}$. This covariance matrix is much smaller than the $mT\times mT$ covariance matrix of the $mT$ individual responses per cluster, hence using cluster-period means speeds up the calculation of the model parameters and their variances, especially so when the number of subjects $m$ per cluster-period is large.

The vector of fixed effects is denoted $\gamma =(\mu ,{\beta }_{2}, \dots , {\beta }_{T},\theta )^{^\prime}$. These fixed effects, along with the variances of the random effects and cluster autocorrelation are estimated using maximum likelihood. The covariance matrix of the fixed effects is

$$var\left(\widehat{\gamma }\right)={\left(\frac{K}{2}\times {X}_{1}^{^\prime}{V}^{-1}{X}_{1}+\frac{K}{2}\times {X}_{2}^{^\prime}{V}^{-1}{X}_{2}\right)}^{-1}$$

where ${X}_{s}$ is the design matrix in sequence $s=1, 2$, given equal allocation of clusters to both sequences.

### Derivation of the optimal design

Optimal design theory (Atkinson et al., 2007; Berger & Wong, 2009) is used to identify the optimal design. In a cluster-randomized crossover trial, the primary interest is in the treatment effect $\theta$, therefore the variance $var(\widehat{\theta})$ of its estimator is used as an optimality criterion. The smallest variance implies the highest design efficiency and hence the highest power for the test on treatment effect. The optimal design is the design ${\xi }^{*}$ among all possible designs in the design space $\Omega$ for which the variance is minimized. The efficiency of any other alternative design $\xi$ in the design space, relative to the optimal design is calculated as

$$RE=\frac{{var(\widehat{\theta })}_{{\xi }^{\ast}}}{{var(\widehat{\theta })}_{\xi }},$$

where the numerator and denominator are the variances obtained with the optimal design and alternative design, respectively. The inverse of $RE$ quantifies how often the alternative design needs to be replicated to perform as well as the optimal design. If $RE=0.9$ then $100\%\left(\frac{1}{0.9}-1\right)=11\%$ additional clusters are needed; if $RE=0.8$ then $100\%\left(\frac{1}{0.8}-1\right)=25\%$ additional clusters are needed. In the remainder of this paper a design with $RE\ge 0.95$ is called highly efficient.

For both aims of this contribution, two different types of design space are taken into account. For the first type, any financial considerations are not of importance. In other words, the design space ${\Omega }_{K}$ is defined to consist of all designs with the same number of clusters $K$. It consists of designs with any number of time periods and any number of treatment switches, as long as $K$ clusters are included. The choice of $K$ is made by the researcher and it is often the number of clusters that are willing to participate in the trial. For the second type, a budgetary constraint is taken into account. The design space ${\Omega }_{B}$ is defined to consist of all designs for which the costs $C$ to enroll subjects and clusters, to implement the treatments and treatment switches, and to measure outcome variables do not exceed the budget $B$ that is available. This design space is thus defined by the budgetary constraint $C\le B$. The amount of budget depends on the amount of funding received for the trial. The costs are calculated as

$$C={c}_{c}K+{c}_{s}KTm+{c}_{x}K(T-1)$$

where ${c}_{c}$ are the costs to recruit one cluster, ${c}_{s}$ are the costs to include, treat, and measure one subject and ${c}_{x}$ are the costs per treatment switch (in either direction). So the costs are a function of the number of clusters, number of treatment switches, and cluster-period size. This cost equation is the same as in Grantham, Kasza, Heritier, Hemming, Litton, et al. (2019b). It should be mentioned that the same equation applies to a cohort design since what matters is how many subjects $m$ there are per cluster-period and not if subjects are included in just one or all time periods. The number of clusters in this type of design space is not fixed beforehand but calculated from the budgetary constraint:

$$K=\frac{C}{{c}_{c}+{c}_{s}Tm+{c}_{x}(T-1)}.$$

So, the number of clusters that fulfill the budgetary constraint is calculated from user-specified costs, budget, and the number of time periods and number of subjects per cluster-period. In other words, the design space ${\Omega }_{B}$ consists of all designs with any number of clusters and treatment switches as long as $K$ is chosen such that the budgetary constraint holds. The value of $K$ should, of course, be an integer and it should be even as well so that both sequences have the same number of clusters. It should be mentioned that the definitions of the design spaces ${\Omega }_{K}$ and ${\Omega }_{B}$ as given above do not depend on whether the first or second research aim is considered.

An analytical expression of the variance of interest, $var(\widehat{\theta })$, is overly complicated and cannot be easily used to study the effect of a certain number or time periods (research aim 1) or the effect of a certain treatment sequence (research aim 2) on design efficiency. For that reason, this variance will be evaluated numerically for all possible designs in the design space ${\Omega }_{K}$ or ${\Omega }_{B}$, for selected values of the intraclass correlation $\rho$, cluster autocorrelation $r$, cluster-period size $m,$ and the three cost components ${c}_{c}$, ${c}_{s}$ and ${c}_{x}$. A restriction is made to at most 12 time periods (e.g., a study with a duration of at most a year and monthly measurements or a study of one quarter with weekly measurements). To facilitate the use of the methodology in this contribution, a Shiny app (Chang et al., 2016) was developed (https://utrecht-university.shinyapps.io/OD_CRXO/). The source code for this app is available online (https://github.com/MirjamMoerbeek/OD_CRXO).

### Motivating example: Intervention to reduce occupational

#### Sedentary behavior

A pilot feasibility study (Nicolson et al., 2021) was used as a motivating example to illustrate the methodology in this paper. This study aimed to assess the acceptability and feasibility of a gender-sensitive multicomponent intervention to reduce occupational sedentary behavior in professional male employees. Two worksites were included in this feasibility study. Worksite A ($n= 8$) started in the intervention condition in time period 1 and switched to the control in time period 2. In worksite B ($n=13$), those two conditions were implemented in reverse order. Both time periods lasted 14 days with a washout period of 7 days in between. Primary outcomes were acceptability and feasibility; secondary outcomes were sedentary behavior, standing, physical activity, work engagement, and anticipated/perceived intervention benefits. Preliminary data showed the intervention may reduce occupational sedentary behavior and increase physical activity. The authors conclude that the intervention should further be tested in a future definitive cluster randomized controlled trial.

Such a definitive trial will include more subjects and worksites than the feasibility study but the number of worksites that may be willing to participate may be limited. Using a crossover design with more than just two time periods may be considered to increase design efficiency. Relevant questions at the design phase may be how many time periods should be included, and how many times a treatment switch should be implemented. To answer these questions, a priori estimates of the intraclass correlation coefficient $\rho$ and cluster autocorrelation coefficient $r$ are needed. Unfortunately, these model parameters cannot be estimated on the basis of a study with just two worksites with eight and 13 participants per worksite. Let us assume $\rho =0.1$ and $r=0.95$. In other words, 10% of the variance in the outcome is located at the cluster level and there is a decay in the correlation of 5% between any two adjacent time periods. Furthermore, we assume in the definitive study $m=25$ participants can be included per cluster-period.

The optimal designs for design space ${\Omega }_{B}$ are derived for ${c}_{c}=2500$, ${c}_{s}=25$, ${c}_{x}=250$, and $B=\text{250,000}$. Costs at the cluster level include incentives to participate, costs to recruit participants, and costs for introduction and presentation sessions. Costs at the subject level include costs to implement the intervention or control and to measure the outcome variables. Costs per treatment switch include costs to collect, clean, store, and/or redistribute equipment such as smartwatches and under-desk pedal machines. The next two sections show how a definitive trial can be designed in an optimal way.

## Research aim 1: Optimal number of time periods

The first research aim of this contribution is to study the optimal number of time periods. Here we focus on designs with a treatment switch after each time period, such as illustrated in Fig. 1. Figure 2 shows the relation between the number of time periods (ranging from 2 to 12) and the relative efficiency, for both design spaces ${\Omega }_{K}$ and ${\Omega }_{B}$.

Using design space ${\Omega}_{K}$, for which the number of clusters $K$ is fixed, the relation between the number of time periods and relative efficiency is almost linear. It is not completely linear since the number of clusters is rounded to an even integer. However, such a relation holds for any number of clusters. The optimal design is found at $t=12$ time periods and a steep decline of the relative efficiency is observed when the number of time periods decreases. Only for $t=11$ the relative efficiency is above 0.9, and at least $t=10$ time periods are needed to achieve a relative efficiency above 0.8. So for this trial configuration, there exist no highly efficient designs with $RE\ge 0.95$.

When a budgetary constraint is taken into account (i.e., design space ${\Omega }_{B}$), then the optimal design is also achieved at $t=11$ time periods. So for this design space adding time periods does not always result in a more efficient design. However, with a relative efficiency as high as 0.984, a design with 12 sequences performs hardly worse than the optimal design. All alternative designs with at least nine time periods are highly efficient. Furthermore, the relative efficiency of alternative designs is higher as compared to the relative efficiency obtained for a fixed number of clusters. For as few as $t=6$ time periods, it is already above 0.8 and for $t=8$ time periods, it is above 0.9. It can further be seen that the relation is clearly non-linear: the benefits of adding a time period are larger for designs with a small number of time periods than for designs with a large number of time periods. For a design with $t=11$ time periods, adding a time period results in a decline in efficiency.

The results in Fig. 2 hold for a particular trial configuration, that is, for one set of values for the cluster-period size $m$, the two model parameters $\rho$ and $r$, the three costs ${c}_{c}$, ${c}_{s}$ and ${c}_{x}$ and budget $B$. To further study the effect of each of these seven quantities on the relative efficiency, three different values for each quantity were studied while fixing the values of the other six quantities equal to those used in Fig. 2. The three selected values for each quantity are given in Table 1; those in boldface are those that were used in Fig. 2.

**Table 1:** Model parameters and their values used in Fig. 3

Parameter	Definition	Values used in Fig. 3
${\varvec{m}}$	Number of subjects per cluster-period	5, 2**5**, 50
${\varvec{\rho}}$	Intraclass correlation coefficient	0.05, **0.1**, 0.15
${\varvec{r}}$	Cluster autocorrelation	0.8, 0.9, **0.95**
${{\varvec{c}}}_{{\varvec{c}}}$	Costs per cluster	1000, **2500**, 5000
${{\varvec{c}}}_{{\varvec{s}}}$	Costs per subject	5, 2**5**, 50
${{\varvec{c}}}_{{\varvec{x}}}$	Costs per crossover	100, **250**, 1000
${\varvec{B}}$	Budget	100000, **250000**, 500000

Values in boldface are used in Fig. 2 as well

**Fig. 3:**
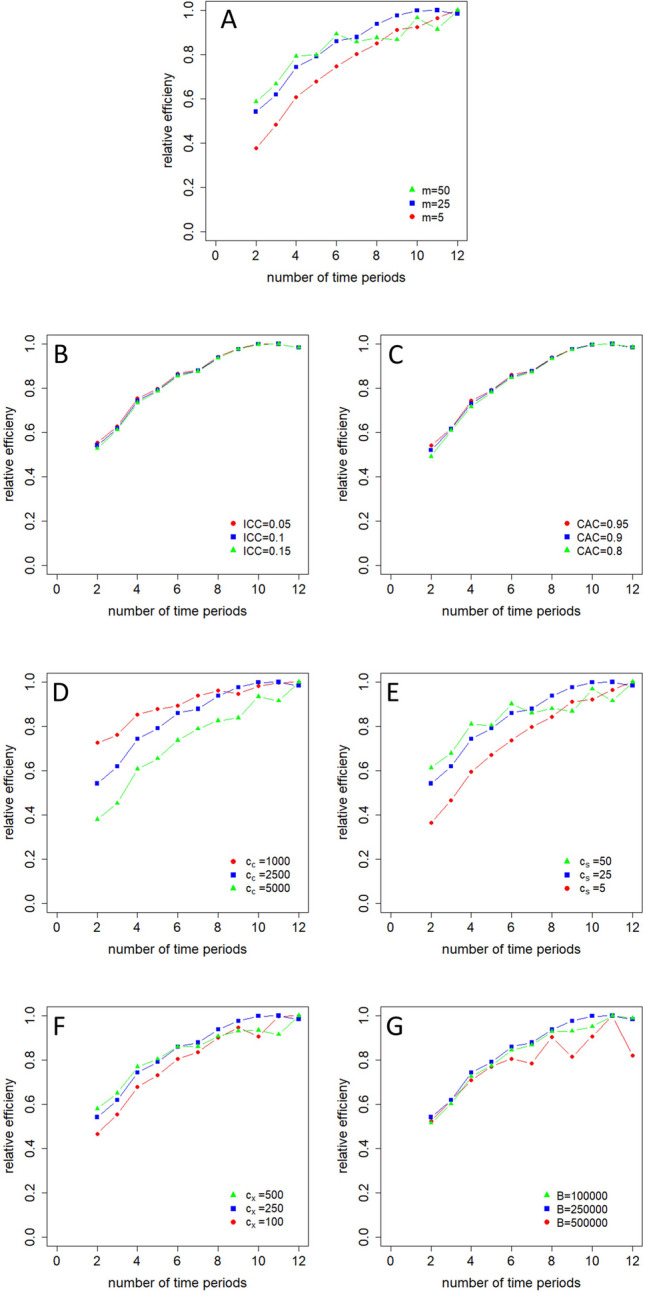
Relation between the relative efficiency and cluster-period size (**A**), intraclass correlation coefficient (**B**), autocorrelation coefficient (**C**), costs per cluster (**D**), costs per subject (**E**), costs per treatment switch (**F**), and budget (**G**)

The results for designs with a fixed number of clusters hardly change when changing the values of these seven quantities; those for designs with a budgetary constraint are displayed in Fig. 3. Panel A shows the relative efficiencies as a function of the number of time periods for various values of the cluster-period size $m$. For $m=5$ or $m=50$ the optimal design has $t=12$ time periods, while for $m=25$ it has $t=11$ time periods. The loss in efficiency of using another design that the optimal one is stronger for $m=5$ than it is for $m=25$. The relation between the relative efficiency and number of time periods is clearly non-monotone for $m=50$. Panel B shows the relative efficiencies as a function of number of time periods for various values of the intraclass correlation coefficient $\rho$. For each chosen value of $\rho$, the optimal design has $t=11$ time periods. Furthermore, the relative efficiencies only slightly depend on $\rho$. Similarly, for each of the chosen values of the autocorrelation $r$ the optimal design has $t=11$ time periods, see panel C, and the effect of $r$ on the efficiencies of the other designs is only minor. Panel D shows how the cluster-level costs ${c}_{c}$ affect the relative efficiencies as a function of the number of time periods. For ${c}_{c}=1000$ and ${c}_{c}=5000$ the optimal design is found at $t=12$ time periods, while for ${c}_{c}=2500$ it is found at $t=11$ time periods. For a number of time periods $t\le 8$ the relative efficiency increases with decreasing ${c}_{c}$, but this is not the case for a larger number of time periods. Panel E shows how the subject-level costs ${c}_{s}$ influence the relative efficiencies as a function of the number of time periods. For ${c}_{s}=5$ and ${c}_{c}=50$, the optimal design is found at $t=12$ time periods, while for ${c}_{c}=25$ it is found at $t=11$ time periods. For a number of time periods $t\le 6$ the relative efficiency increases with increasing ${c}_{c}$, but this is no longer the case for larger number of time periods. Panel F shows a rather similar pattern for various values of the costs ${c}_{x}$ of a treatment switch. For ${c}_{x}=100$ and ${c}_{x}=500$ the optimal design is found at $t=12$ time periods, while for ${c}_{x}=250$ it is found at $t=11$ time periods. For the number of time periods $t\le 5$, the relative efficiency increases with increasing ${c}_{c}$, but this is not the case for a larger number of time periods. Finally, panel G shows that for each of the three chosen budgets $C$, the optimal design has $t=11$ time periods. For the number of time periods $t\le 5$ the relative efficiency hardly depends on the budget $C$, while for a larger number of time periods the loss in efficiency of not using the optimal design is largest for $C=\text{500,000}$.

## Research aim 2: Optimal number of treatment switches

The second research aim of this contribution is to study if a treatment switch has to be made at the end of each time period. When the costs of a switch are high relative to other costs, a design in which clusters stay in the same treatment for a few consecutive time periods may be more efficient than a design with a switch at the end of each time period.

**Fig. 4:**
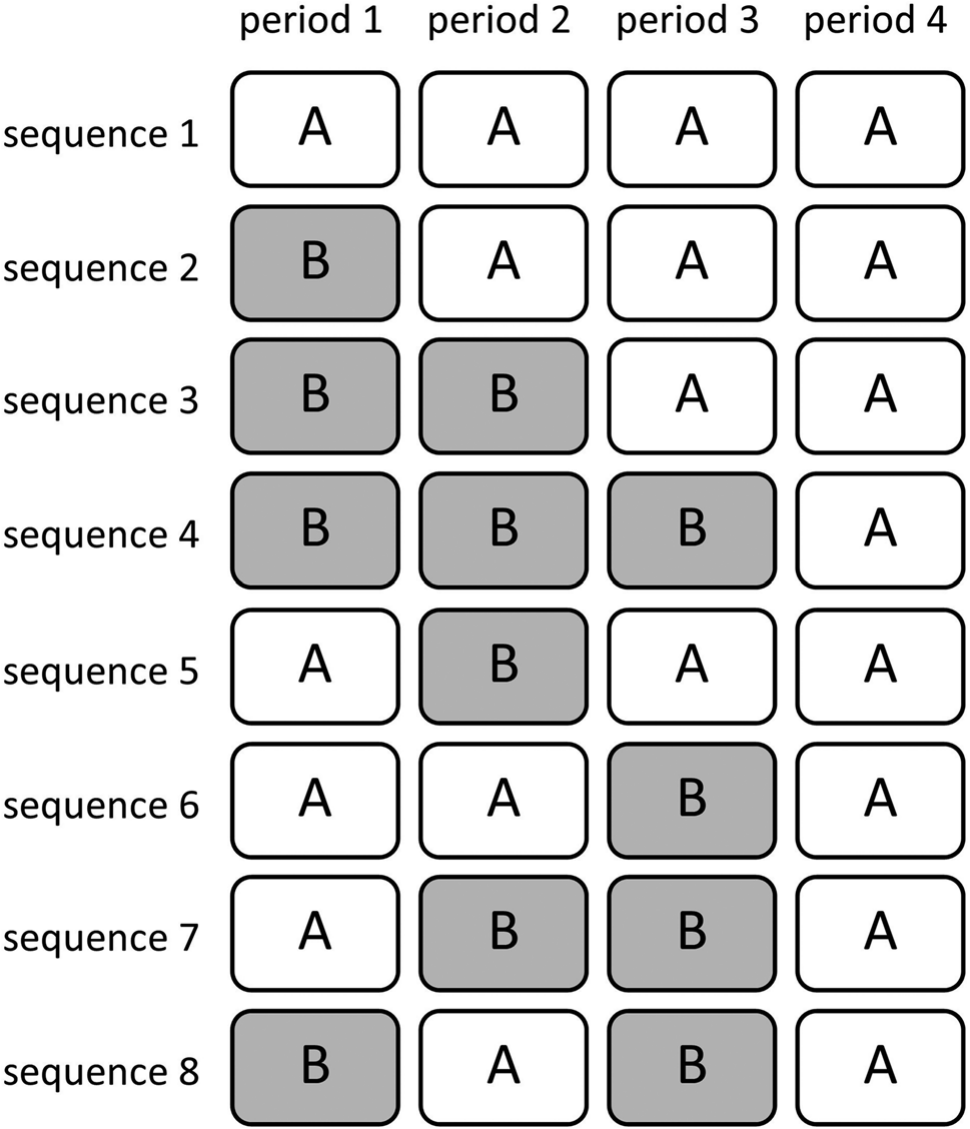
An overview of possible treatment sequences in a cluster randomized crossover trial with four time periods. One half of the sequences is given, and the other half is the mirror of this half

Consider as an example a trial with $T=4$ time periods. Then the maximum number of sequences is ${2}^{4}=16$. However, we restrict to designs with two sequences, where one sequence is a mirror of the other sequence. In other words, when clusters in one sequence receive treatment A in a particular time period, then clusters in the other sequence receive treatment B in the same time period, and vice versa. Eight sequences are given in Fig. 4, the other eight sequences are their mirrors and are not included in this figure. In sequence 1, there is no treatment switch at all, implying the design is a parallel-group cluster randomized trial. Sequences 2–4 have only one switch, and they differ in their timing of that switch. In sequence 3, both treatments are offered in two consecutive time periods, while in sequences 2 and 4 one treatment is offered in one time period and the other in three consecutive time periods. Sequences 5–7 have two treatment switches, and also they differ with respect to the timing of their switches. In sequence 7, both treatments are offered in two time periods (although not in consecutive time periods for treatment A), while in sequences 5 and 6 the one treatment is offered in three time periods and the other in only one. Finally, sequence 8 has a maximum of three treatment switches, one after each time period.

Table 2 gives a comparison of the eight treatment sequences in terms of their relative efficiency. The same parameter values and costs as in the previous section have been used. The column labeled ${RE}_{K}$ is the relative efficiency if the number of clusters is fixed beforehand. The results in this column do not depend on the number of clusters that are included in the trial. Sequence 8, which has a maximum of three treatment switches, performs best. The second best choice is sequence 7, which has 2 treatment switches and is highly efficient with ${RE}_{K}=0.985$. This sequence performs better than the other two sequences with two treatment switches (sequences 5 and 6). The most likely explanation of these results is that in sequence 7 both treatments are available in two time periods. So in this treatment sequence, both conditions contribute equally to the within-cluster treatment comparison, resulting in a higher efficiency than that of designs with one treatment in one time period and the other sequence in three time periods. Sequences 5 and 6 perform equally well because of their centrosymmetry in terms of the timing of their treatment switches: in sequence 5 the treatments are offered in the reverse order as in sequence 6. The third choice is sequence 3, which only has one treatment switch and ${RE}_{K}=0.811$. This sequence outperforms sequences 5 and 6, which have two switches each. Sequence 3 also outperforms sequences 2 and 4, which also have one switch. The most likely explanation of these results is that in treatment 3 both treatments A and B are available in two time periods, which is not the case for sequences 2, 4, 5, and 6. Sequence 7 outperforms sequence 3, even though for both of them both treatments are available in two time periods. This is most likely because in sequence 7 there are more treatment switches than in sequence 3. Sequences 2 and 4 perform equally well because of their centrosymmetry. Sequences 1, 2, 4, 5, and 6 all have a relative efficiency below 0.8, meaning at least 25% extra clusters are needed to perform as well as the optimal sequence 8. Sequence 1, which does not have any switches at all, is not recommended since it has a very low relative efficiency.

**Table 2:** Relative efficiency of all possible sequences in a trial with four time periods, for a fixed number of clusters (${RE}_{K}$) and for a budgetary constraint (${RE}_{B}$)

Sequence	Order of treatments	Number of switches	${RE}_{K}$	*K* For budgetary constraint	Costs For budgetary constraint	${RE}_{B}$
**1**	AAAA	0	0.098	50	250,000	0.114
**2**	BAAA	1	0.672	46	241,500	0.715
**3**	BBAA	1	0.811	46	241,500	0.863
**4**	BBBA	1	0.672	46	241,500	0.715
**5**	ABAA	2	0.775	44	242,000	0.787
**6**	AABA	2	0.775	44	242,000	0.787
**7**	ABBA	2	0.985	44	242,000	1
**8**	BABA	3	1	42	241,500	0.967

Trial configuration with intraclass correlation coefficient $\rho =0.1$, cluster autocorrelation $r=0.95$, $m=25$ subjects per cluster-period, costs ${c}_{c}=2500$, ${c}_{s}=25$ and ${c}_{x}=250$ and budget $B=\text{250,000}$

The last three columns in Table 2 are for trials that take a budgetary constraint into account. The sample size and costs depend on the number of treatment switches, but not on the timing of these switches. The costs may be somewhat lower than the budget since the number of clusters is rounded downwards to an even integer. For the same reason, the costs of the designs in this table are slightly different from each other. As is obvious, fewer clusters $K$ can be included in the trial when more switches are included. For design 1, the costs are equal to the available budget, for all other designs the costs are slightly lower. The column labeled ${RE}_{B}$ gives the relative efficiencies of the eight sequences under a budgetary constraint. Apparently, the costs of a switch are such high that sequence 8 is no longer the optimal one. It is then better to implement one switch less than the highest possible number of switches and use the thus saved budget to include more clusters. However, the relative efficiency of design 8 is ${RE}_{B}=0.967$, meaning it is a highly efficient alternative to the optimal design 7. The third choice is sequence 3, which only has one treatment switch. Sequence 1, which has no switches at all, is clearly not recommended. Again we observe that designs that are centrosymmetric (designs 2 and 4, and designs 5 and 6 ) have equal relative efficiencies. Furthermore, design 3, in which treatments A and B are both included in two time periods, again outperforms designs 1, 2, 4, 5, and 6.

The reader is invited to assess the sensitivity of these results against different values of the model parameters, costs, and budget using the Shiny app. For trials with a fixed number of clusters, he or she may notice that the design with the maximum number of switches is optimal. For designs with a budgetary constraint, he or she may notice that the costs of a switch have an effect on the optimal number of switches. For instance, when these costs increase to ${c}_{x}=1000$ then not only sequence 7 (with two switches) but also sequence 3 (with only one switch) outperforms sequence 8 (with three switches). On the other hand, when the cost of a switch is as low as ${c}_{x}=50$, then sequence 8 is the optimal one. So the costs of a switch determine if the sequence with the maximum number of switches is indeed the optimal one or if other sequences with fewer switches are preferred. Also, the number of subjects per cluster-period has a clear effect on the results. With only five subjects per cluster-period, sequence 3 (with only one switch) is the optimal one. With 25 subjects per cluster-period, sequence 7 (with two switches) is the optimal one. With as many as 50 subjects per cluster-period, sequence 8 (with three switches) is the optimal one. So in this trial configuration, the optimal number of switches increases with the number of subjects per cluster-period.

## Discussion

When the number of clusters is fixed a priori, then including an extra time period makes the trial more efficient. This makes sense because adding time periods implies adding subjects to the trial, and it is well known that a larger sample size results in a more efficient design. Furthermore, for any chosen number of time periods, the design with a treatment switch at every time period is the most efficient. As an exponential correlation structure is used, subjects who are closer together in time are more alike. In a cluster randomized crossover trial, the estimation of the treatment effect follows from within-cluster comparisons. Therefore, when a treatment switch is made at each time period, then comparisons between treatments are made on the basis of subjects who are closer in time, and hence more similar. It therefore makes sense that more treatment switches result in a more efficient design.

These results do not necessarily hold when a budgetary constraint is taken into account. The optimal design and the efficiency of alternative designs are then determined by the costs per cluster, per subject and per treatment switch, the number of subjects per cluster-period, and the intraclass and autocorrelation coefficients. When treatment switches are relatively expensive, then a design with fewer than the maximum number of time periods may be optimal, as was demonstrated in Fig. 2. Furthermore, it may also turn out that it may not be necessary to implement a switch at each time period.

When studying the optimal number and timing of treatment switches, it was observed that designs that are centrosymmetric in terms of the timing of their treatment switches perform equally well. Furthermore, a higher number of treatment switches does not necessarily result in a more efficient design. A sequence with fewer switches but with both treatments in the same number of time periods may perform better than a sequence with more treatment switches and the one treatment in more time periods than the other. Furthermore, design efficiency not only depends on the number and timing of treatment switches but also the values of the intraclass and cluster autocorrelation coefficients, the cluster-period size, and the costs and budget. Therefore it is very complicated to give general theoretical results of why and under which conditions certain treatment sequences outperform others. The Shiny app can be used to determine the optimal design for a trial at hand and to evaluate the efficiency of alternative designs for a specific trial configuration.

This app may also be used to assess the sensitivity of the optimal design against the uncertainty of the intraclass and autocorrelation coefficients. The values of these model parameters may often be unknown in the design phase of a trial and an educated guess may be based on the literature or expert knowledge. The online CLOUDbank summarizes estimates obtained from empirical data for various types of clusters (Korevaar et al., 2021). One may also select a range, rather than a point estimate, of these two model parameters and study if the optimal design changes a lot when alternative values within these ranges are chosen. For cluster randomized trials with a single time period and a budgetary constraint, it has already been shown that the optimal design is rather robust against incorrect a priori estimates of the intraclass correlation coefficient (Korendijk et al., 2010). Future research will have to show if this is also the case for crossover trials.

Another direction for future research is the effect of attrition on the optimal design. Attrition is the rule rather than the exception in longitudinal designs such as the multiperiod cluster randomized crossover trial. When the duration of a trial is long, it may very well occur clusters drop out, for instance, because they lose interest in the study or no longer have the means to participate. The effects of attrition have already been studied for the two-period cluster randomized crossover trial (Moerbeek, 2020) and for the multiperiod cluster randomized trial with a parallel group design (Moerbeek, 2021), and future research will focus on the multiperiod crossover design. A related issue is the possibility of unequal cluster-period sizes within and between clusters. It may very well occur that it is more difficult to recruit eligible subjects in the one time period than in the other, or in the one cluster than in the other. Furthermore, subjects may drop out during the course of their treatment, which also results in unequal cluster-period sizes. It has already been shown that unequal cluster sizes result in a decline of efficiency on cluster randomized trials with a single time period (Van Breukelen et al., 2007), and future research may focus on multiperiod cluster randomized trials.

It should finally be mentioned that the derivation of the optimal design is done under the assumption that the data that are collected during the trial will be analyzed using the multilevel regression model. This model includes clusters as random effects, so that the results of the study may be generalized to the population from which these clusters are sampled. The number of clusters should be sufficiently large so that the clusters are representative for their population; it has been suggested to use at least 10 to 20 clusters (Snijders & Bosker, 2012). For a smaller number of clusters, the random cluster effects should be replaced by fixed effects (i.e., using dummy variables to represent the clusters). The optimal design methodology in this paper is therefore not recommended for trials with fewer than 20 clusters. Future research should focus on optimal designs for trials with a smaller number of clusters.

## Conclusion

This contribution has shown that a design for a cluster randomized crossover trial with as many time periods and treatment switches as possible is not necessarily optimal in the case a budgetary constraint is taken into account. For each trial at hand, the Shiny app may be used to determine the optimal design and efficiency of alternative designs. To my knowledge, this was the first study on the optimal number of time periods and treatment switches for cluster randomized trials in the case the duration of the time periods is fixed. I hope researchers may find the methodology in this contribution useful for planning their own future trials.

## Data Availability

Data sharing is not applicable to this article as no datasets were generated or analyzed during the current study.

## Footnotes

1 If ${\beta }_{1}\ne 0$ then the columns in the design matrices ${X}_{s}$ for sequences s = 1,2 become a linear combination of each other. This implies the model’s fixed effect parameters $\mu$, ${\beta }_{1}$ and $\theta$ cannot be estimated.

## Code availability

The code underlying the Shiny app can be found at https://github.com/MirjamMoerbeek/OD_CRXO
